# UAV-based disease detection in strawberry by multispectral image segmentation invoking deep learning

**DOI:** 10.18699/vjgb-26-97

**Published:** 2026-10

**Authors:** A.F. Cheshkova, V.S. Riksen, O.V. Elkin

**Affiliations:** Siberian Federal Scientific Center of AgroBioTechnologies of the Russian Academy of Sciences, Krasnoobsk, Novosibirsk region, Russia; Siberian Federal Scientific Center of AgroBioTechnologies of the Russian Academy of Sciences, Krasnoobsk, Novosibirsk region, Russia; Siberian Federal Scientific Center of AgroBioTechnologies of the Russian Academy of Sciences, Krasnoobsk, Novosibirsk region, Russia

**Keywords:** strawberry, leaf spot disease, UAV, multispectral imaging, deep learning, U-Net, semantic segmentation, precision agriculture, земляника садовая, белая пятнистость, БПЛА, мультиспектральная съемка, глубокое обучение, U-Net, семантическая сегментация, точное земледелие

## Abstract

Accurate and timely diagnosis of strawberry diseases is crucial for minimizing crop loss and reducing the cost of plant treatment. The use of unmanned aerial vehicles (UAVs) equipped with multispectral cameras opens up new possibilities for noninvasive plant health monitoring. The objective of this research is to explore the feasibility of detecting diseases in strawberries through multispectral imagery obtained from UAVs combined with deep learning techniques. Leaf spot disease, caused by Ramularia tulasnei Sacc., was chosen as a case study. A DJI Phantom 4 Multispectral quadcopter was used to survey an experimental plot (37 × 22 m) from an altitude of 3 m, achieving a resolution of 2.2 mm per pixel. Preprocessing of the acquired data involved orthomosaic building using Agisoft Metashape Pro and manual region-of-interest (ROI) extraction in ENVI 5.2. A dataset comprising 360 multispectral images, each of size 64 × 64 × 5 (with spectral bands at 450, 560, 650, 730, and 840 nm), was created and separated into three classes: healthy plants, diseased plants, and soil. Using this dataset, a U-Net-based model – supplemented with the attention gate, squeeze-and-excitation, and residual connections modules – was trained. Additionally, to assess the contribution of multispectral channels, another U-Net model was trained on RGB images, as well as a classic Random Forest model based on 40 GLCM texture features. The U-Net model trained on multispectral data achieved an overall classification accuracy of 96.04 %, which is significantly higher than the performance of Random Forest (76.64 %) and the U-Net on RGB images (94.14 %). The resulting disease distribution maps enable site-specific fungicide application. The proposed approach confirmed the effectiveness of deep learning for diagnosing leaf spot disease in strawberries. Future research will focus on expanding the dataset (different cultivars and disease stages), testing more advanced segmentation architectures, and scaling the method to commercial plantations.

## Abstract (ru)

Точная и своевременная диагностика болезней земляники садовой является важной задачей, позволяющей минимизировать потери урожая и снизить затраты на обработку растений. Использование беспилотных летательных аппаратов (БПЛА) с мультиспектральными камерами открывает новые возможности для неинвазивного мониторинга состояния растений. Цель настоящего исследования – анализ возможности выявления заболеваний земляники садовой (на примере белой пятнистости, возбудитель Ramularia tulasnei Sacc.) по мультиспектральным изображениям, полученным с БПЛА, с использованием методов глубокого машинного обучения. Съемка опытного участка (37 × 22 м) проводилась квадрокоптером DJI Phantom 4 Multispectral на высоте 3 м (разрешение 2.2 мм/пиксель). Полученные данные прошли предварительную обработку, включая построение ортофотоплана в программе Agisoft Metashape Pro и ручное выделение областей интереса (ROI) в ENVI 5.2. Сформирован набор из 360 мультиспектральных изображений размером 64 × 64 × 5 (каналы 450, 560, 650, 730, 840 нм) с тремя классами: здоровые растения, больные растения и почва На данном наборе была обучена модель архитектуры U-Net (с модулями attention gate, squeeze-and-excitation и residual connections). Дополнительно для оценки вклада мультиспектральных каналов обучены U-Net модель на RGB-изображениях, а также классическая модель Random Forest на основе 40 текстурных признаков GLCM. Модель U-Net, обученная на мультиспектральных данных, достигла общей точности классификации 96.04 %, что существенно выше показателей Random Forest (76.64 %) и U-Net на RGB-изображениях (94.14 %). Полученные карты распространения болезни позволяют проводить точечное применение фунгицидов. Предложенный подход подтвердил эффективность глубокого обучения для диагностики белой пятнистости земляники. Дальнейшие исследования будут направлены на расширение набора данных (разные сорта и стадии болезней), апробацию более современных архитектур сегментации и масштабирование метода на промышленные плантации.

## Introduction

Crop health monitoring using unmanned aerial vehicles (UAVs) is a key technology in modern precision agriculture (Radoglou-Grammatikis et al., 2020). The potential of UAVs has been demonstrated in a number of applications, such as plant stress detection, field mapping, biomass estimation, weed control, and plant population counting (Toscano et al., 2024). High efficiency, stability, accuracy, and productivity are the advantages of UAVs, enabling farmers and experts to make more informed and timely management decisions (Phang et al., 2023).

The use of various types of sensors mounted on UAVs allows farmers to monitor multiple aspects of plant con-dition.

For example, RGB and near‑infrared images enable evaluation of organ and whole‑plant morphology, growth dynamics, biomass accumulation, yield traits, and pest/ disease severity (Cheshkova, 2022; Zahir et al., 2022). Chlorophyll fluorescence imaging helps the assessment of plant bioenergetic status and genetic stress tolerance (Moustakas et al., 2021). Thermal infrared imaging is used to measure plant temperature, water status, and insect damage (Hou et al., 2022). Laser scanning and 3D imaging are applied to determine seed morphology, leaf orientation, canopy structure, root architecture, and linear plant dimensions (Jin et al., 2021).

One of the important and common tasks in field monitoring is the detection of plant diseases in images acquired by UAVs (Neupane, Baysal-Gurel, 2021). Among UAVmounted sensors, multispectral cameras are the primary tool for disease identification. The spectral data from these cameras enable the computation of various vegetation indices (VIs) that reflect the biophysical and biochemical properties of plants affected by stress (Candiago et al., 2015). Vegetation indices allow quantitation of these changes and are an important factor for identifying crop diseases (Mahlein et al., 2013).

Use of multispectral data goes beyond just calculating spectral indices. A more powerful and precise way to analyze images is through machine learning. These techniques can be broadly split into two categories.

In traditional machine learning methods (e. g., SVM, Random Forest, KNN), an expert manually creates and selects features from raw data (Behmann et al., 2015; Singh et al., 2016). The quality of the model is strongly dependent on the quality of these features. Despite their advantages, these algorithms can only be applied to tabular data, which limits their utility for detecting plant diseases in images or videos (Benos et al., 2021).

Deep learning methods (U‑Net, SegNet, ResNet), in contrast, perform automatic feature extraction. The neural network learns feature hierarchies directly from raw data without manual intervention (Mudarisov, Miftakhov, 2024; Sajitha et al., 2024). These methods can process images and videos to detect and classify plant disease symptoms (Loey et al., 2020; Nagaraju, Chawla, 2020; Abade et al., 2021).

The main tasks in machine‑learning‑based image analysis are segmentation, classification, and detection (Saleem et al., 2019; Ding et al., 2024).

Detecting plant stress from UAV imagery is a semantic segmentation problem: each pixel is labeled as soil, healthy plant, or diseased plant, resulting in a field‑wide map of disease spread.

Among berry crops, strawberry (Fragaria × ananassa) holds the leading position in global production and consumption, accounting for over 70 % of the world’s berry production (Ozherelev et al., 2019). Precise, rapid, and noninvasive detection of diseases and abiotic stress in strawberries is essential for commercial production, enabling reduced crop losses and lower plant management costs (Zheng et al., 2021).

The aim of this study is to analyze the feasibility of detecting diseases in strawberries (Fragaria × ananassa) using multispectral images acquired by UAVs and deep learning methods.

## Materials and methods

Plant material. The research was carried out on strawberry plants growing in an experimental plot of the Siberian Federal Research Center of Agro-BioTechnologies, Russian Academy of Sciences (Krasnoobsk, Novosibirsk region, Russia). The 37 × 22 m plot contained three‑year‑old cv. Festivalnaya plants planted in 50-cm wide rows spaced 70 cm apart. At the time of imaging, most plants were affected by leaf spot disease (caused by Ramularia tulasnei Sacc.) Through visual inspection, 80 healthy plants and 80 plants with visible leaf lesions were selected. The selected plants were tagged with pink and blue labels, respectively (Fig. 1).

**Fig. 1.**
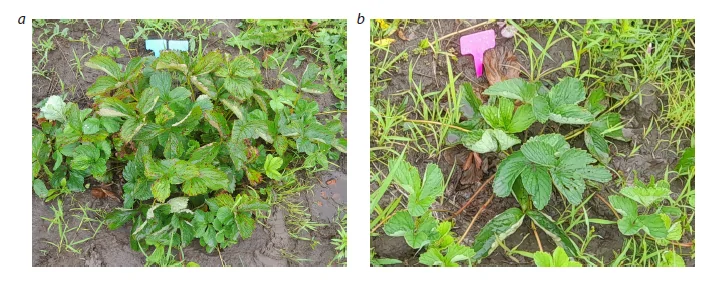
Strawberry plants: a – affected by leaf spot disease; b – healthy.

Image acquisition. A DJI Phantom 4 Multispectral quadcopter (SZ DJI Technology Co., Ltd., Shenzhen, China) was used for image acquisition. Its camera has one RGB (visible light) sensor and five monochrome sensors (blue, green, red, red edge, near‑infrared) at wavelengths of 450, 560, 650, 730, and 840 nm, respectively.

The flight took place on July 15, 2025, under cloudy skies at an altitude of 3 m, yielding a resolution of 2.2 mm/pixel. The mission produced 2,170 images with 75 % forward overlap and 60 % side overlap.

Image preprocessing and dataset generation. Monochrome multispectral cameras capture each spectral band using a separate sensor at different points in time. Due to drone movement and vibrations, these channels are not pixel‑aligned in the raw frames. Prior to model training, it is necessary to bind all spectral channels to a common coordinate system and create a multispectral raster in which each pixel contains all the required wavelengths. This step is mandatory for a neural network being able to correctly extract spectral features; otherwise, pixels from different channels would correspond to different points on the ground.

The first step in processing the images acquired by the five monochrome PM4 cameras was to construct a unified multispectral image (orthomosaic) of the experimental plot. For this purpose, the Agisoft Metashape Pro software (Agisoft LLC, Russia) was used. The final orthomosaic was output as a GeoTIFF file with a resolution of 15,000 × 20,000 × 5 pixels and a size of 2.9 GB.

The second step was image labeling. Axis‑aligned rectangular regions of interest (ROIs) of 64 × 64 × 5 pixels in size were manually drawn on the resulting orthomosaic. These ROIs contained tagged diseased plants, tagged healthy plants, and soil areas (the tags themselves were not included in the ROIs). Each ROI was saved as a separate image file (Fig. 2). ROI extraction and image generation were carried out in ENVI 5.2 (ITT Visual Information Solutions).

**Fig. 2.**
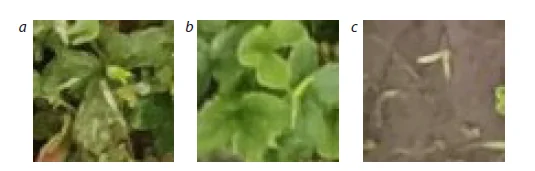
ROI extraction: a – strawberry plants affected by leaf spot disease; b – healthy plants; c – soil.

A dataset of 360 multispectral images (120 per class) was generated in this way: 240 for training, 60 for validation, and 60 for testing.

To enable model accuracy comparisons, a second, RGBbased, dataset was prepared. Here, ROIs were drawn directly on raw PM4 RGB camera images, without orthomosaic generation. The RGB dataset also comprised 360 images of size 64 × 64 × 3.

For comparison with classical machine learning, an additional tabular dataset was created as follows. For each training multispectral image (64 × 64 × 5), eight GLCM‑based texture features (mean, variance, homogeneity, contrast, dissimilarity, entropy, second moment, and correlation) were computed pixel‑wise per channel (Li et al., 2019; Guo et al., 2021; Zhou et al., 2022) using a sliding window of (3×3) px.

For pixels whose local neighborhood extended beyond the image boundaries, the texture feature values were set to NA (not available). The relative distance measured in pixels (d) in the GLCM was set to 1 (d = 1). Matrices were computed for four directions (Θ = 0°, 45°, 90°, 135°), and the feature values were averaged across these directions. Each image yielded a table with 4,096 rows (64 × 64), 40 feature columns (8 features × 5 bands), and a class column (healthy, diseased, soil). From each table, 1,000 pixels were randomly sampled. The final training set had 240,000 rows (80,000 per class), with validation and test sets of 60,000 rows each.

Modeling and data analysis methods. All calculations and data analysis were performed in R environment using the torch, geodl, randomForest, and terra packages.

Two machine learning models were considered in this study: the U‑Net deep learning model (Ronneberger et al., 2015) and the classical Random Forest model (Breiman et al., 2001).

The U‑Net convolutional neural network is one of the most popular architectures for solving semantic segmentation tasks (Kerkech et al., 2020; Zhang T. et al., 2021). The U‑shaped architecture of the network, consisting of a downsizing path (encoder) for capturing context and a symmetrically upsizing path (decoder) for precise localization, along with skip connections between the encoder and decoder, enables the network to combine high‑level features with detailed spatial information. This method produces highly accurate segmentation maps even when training data are limited.

We used the U‑Net architecture shown in Figure 3, implemented in the geodl package (Maxwell et al., 2024). This architecture can accept a variable number of input predictor variables, making it suitable for both RGB and multispectral images. The architecture comprises four encoding blocks, one bottleneck block, and four decoding blocks. The number of output feature maps was set to (16, 32, 64, 128) in the encoding blocks and (128, 64, 32, 16) in the decoding blocks. Rectified linear unit (ReLU) activation functions were implemented throughout the architecture to incorporate nonlinearity, and batch normalization was used to combat gradient issues and aid in convergence.

**Fig. 3.**
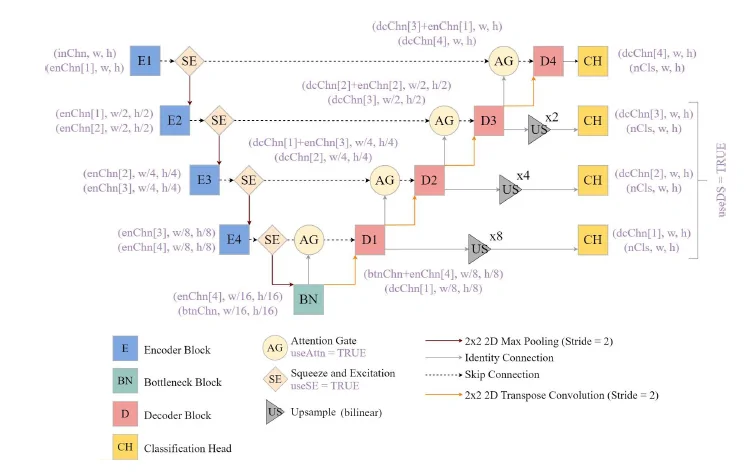
U-Net architecture implemented in geodl. Blocks prefixed with E represent encoder blocks while those prefixed with D represent decoder blocks; w – width, h – height.

In the encoder, the size of the array in the spatial dimensions is reduced by half following each block using 2 × 2 max pooling with a stride of 2. In the decoder, 2D transpose convolution is used to double the spatial resolution of the feature maps provided from the prior block, also using a stride of 2. The final class logits are predicted using 1 × 1 2D convolution. Attention gate, squeeze-andexcitation, and residual connection modules were added to the main blocks of the U-Net architecture. 

The classical Random Forest model, which is based on an ensemble of decision trees, is also frequently applied to remote sensing tasks, including the detection and classification of plant diseases (Lan et al., 2020; Liao et al., 2022; Zhang S. et al., 2022). In our previous work, we found that for detecting strawberry diseases from multispectral images using Random Forest, texture features perform best as predictors (Cheshkova, Riksen, 2025).

Model performance metrics. Segmentation quality was evaluated using standard per‑class metrics: Accuracy, Precision, Recall, F1-score и IoU. Their calculation is based on the elements of the confusion matrix: TP (true positive) is the number of pixels belonging to the class under consideration that were correctly assigned to this class by the model; TN (true negative) is the number of pixels belonging to all other classes that were correctly not assigned to the class under consideration; FP (false positive) is the number of pixels from other classes that were incorrectly assigned to the class under consideration; and FN (false negative) is the number of pixels belonging to the class under consideration that were incorrectly assigned to other classes.

Accuracy (the proportion of correct classifications) represents the proportion of all pixels that were classified correctly when evaluating a particular class using a onevs- rest scheme. It accounts for both the pixels of the class under consideration that were correctly identified and the pixels correctly assigned to all other classes. The closer the Accuracy value is to 1 (100 %), the higher the classification accuracy.

**Accuracy:**
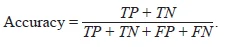
Accuracy

Precision indicates the proportion of pixels assigned by the model to the class under consideration that actually belong to that class. A high Precision value corresponds to a low number of false-positive classifications.

**Precision:**
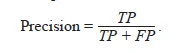
Precision

Recall (sensitivity) characterizes the proportion of pixels belonging to the class under consideration that were correctly identified by the model among all pixels actually belonging to that class. A high Recall value indicates a low number of false-negative results, i. e., few missed pixels of the class under consideration.

**Recall:**
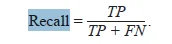
Recall

The F1-score is the harmonic mean of Precision and Recall and is used to assess these two metrics jointly. A high F1-score can only be achieved when both Precision and Recall are high; therefore, this metric accounts for both false-positive and false-negative errors.

**F. 1.**
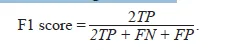
F1

IoU (Intersection over Union) characterizes the degree of overlap between the class mask predicted by the model and the reference mask. The numerator represents the area of intersection between the predicted and reference masks, while the denominator represents the area of their union. An IoU value close to 1 indicates a high degree of spatial agreement between the predicted segmentation and the reference annotation.

**IoU:**
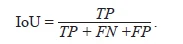
IoU

Overall Accuracy (OA) evaluates classification performance across all classes simultaneously and is defined as the ratio of the total number of correctly classified pixels from all classes to the total number of pixels. Unlike Accuracy, which is calculated separately for each class, Overall Accuracy provides a single summary measure of multiclass image classification performance.

## Results

**Model training and validation**

U‑Net models were trained using the Adam optimizer and CrossEntropyLoss. The training involved Z‑score normalization and data augmentation (horizontal/vertical flip with 0.5 probability, random rotation by 0°, 90°, 180°, or 270°). Models were run for 50 epochs with an initial learning rate of 0.001.

Two training datasets were compared: multispectral and RGB images. Figures 4 and 5 illustrate the U‑Net training dynamics for each dataset.

**Fig. 4.**
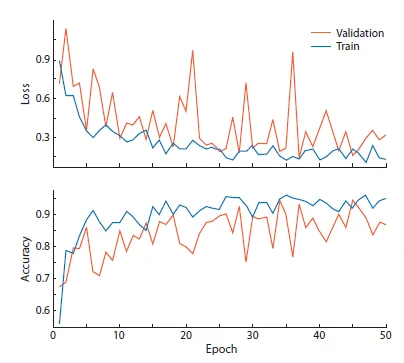
U-Net training accuracy and loss curves on multispectral images.

**Fig. 5.**
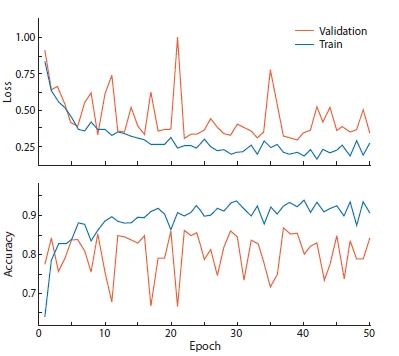
U-Net training accuracy and loss curves on RGB images.

The Random Forest model was trained with the following hyperparameters: number of trees = 500, minimum terminal node size = 1, and number of candidate variables per split = 6.

The trained models were evaluated on the test sets. Tables 1–3 summarize the quality metrics.

**Table 1.**
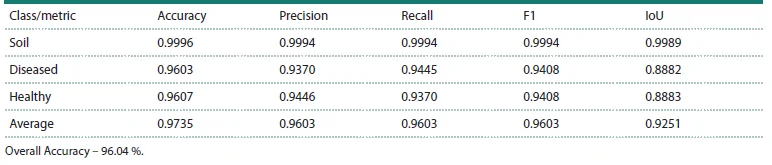
Performance metrics of the U-Net model trained on multispectral images

**Table 2.**
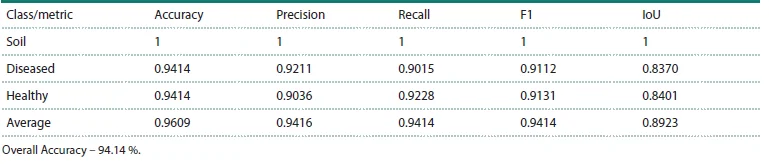
Performance metrics of the U-Net model trained on RGB images

**Table 3.**
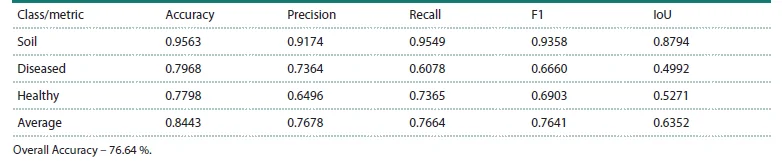
Performance metrics of the Random Forest model trained on texture features of multispectral images

The overall classification accuracy of the U‑Net model trained on multispectral imagery reached 96.04 %. A slightly lower accuracy was observed for the U‑Net model trained on RGB imagery – 94.14 %. In contrast, the Random Forest model performed considerably worse at 76.64 %.

The trained models were then applied to segment the images and produce disease distribution maps for the experimental strawberry plot (Fig. 6).

**Fig. 6.**
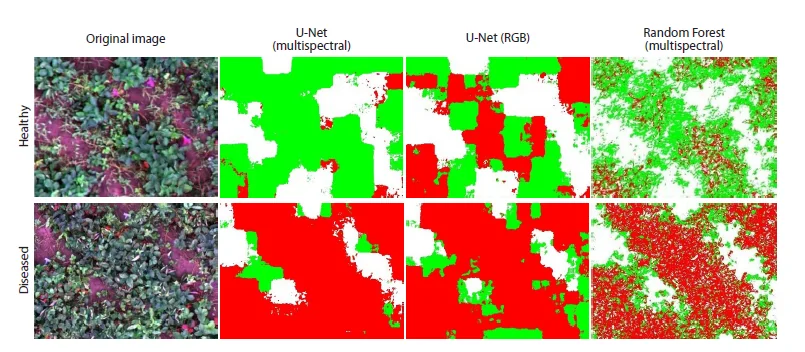
Image segmentation results from the trained models. Green – healthy plants, red – diseased plants, white – soil.

## Discussion

This study evaluated the feasibility of detecting diseases in strawberries (with leaf spot as an example) from UAV‑acquired multispectral imagery using the U‑Net deep learning architecture and the traditional Random Forest method. The results obtained demonstrated that the U‑Net model trained on multispectral data (five channels at 450, 560, 650, 730, and 840 nm) achieved an overall classification accuracy of 96.04 %, surpassing the accuracy of the Random Forest model (76.64 %) as well as that of the U‑Net model trained solely on RGB images (94.14 %).

The accuracy achieved by our U‑Net model (96.04 %) is comparable to, or exceeds, the results reported in other studies on crop disease detection using UAVs and deep learning. For instance, J. Su et al. (2020) and T. Zhang et al. (2021) achieved accuracies of 91 and 97 %, respectively, in detecting yellow rust in wheat using modified U‑Net architectures. M. Kerkech et al. (2020) achieved 85–94 % accuracy with SegNet, DeepLabv3+, and PSPNet for grapevine diseases. Thus, our model lies at the upper end of this range, confirming the suitability of U‑Net for phytopathological tasks in strawberry cultivation.

The multispectral U‑Net outperformed the RGB version (96.04 vs. 94.14 %) because the additional red‑edge (730 nm) and near‑infrared (840 nm) bands are sensitive to changes in chlorophyll, mesophyll structure, and leaf water content – changes that occur during early fungal infection before visible spots appear. This inference agrees with A.-K. Mahlein et al. (2013) and S. Candiago et al. (2015), who emphasized the value of nonvisible spectral bands for early stress detection.

The Random Forest model, built on 40 GLCM texture features, achieved only 76.64 % accuracy. This is much lower than what other researchers have achieved with Random Forest for plant diseases: 90 % for eucalyptus disease (Liao et al., 2022) and 97 % for banana Fusarium wilt (Zhang S. et al., 2022). We attribute this to the specific symptomatology of leaf spot in strawberries: the disease produces small (1–5 mm) necrotic lesions on leaves that do not lead to a global change in the color or texture of the entire leaf. By contrast, banana Fusarium wilt and eucalyptus disease cause massive discoloration of whole leaves, obvious from GLCM features at the pixel‑neighborhood level. GLCM features calculated for a local window (in our case, around each pixel) proved insufficiently sensitive to small‑scale, scattered lesions.

These results clearly show that deep learning with multispectral UAV images is effective for detecting strawberry white leaf spot. U‑Net outperformed both Random Forest and RGB‑only models, confirming the value of nonvisible spectral bands and automatic feature extraction architectures.

Suggested future research directions: • enlarge the dataset with more varieties, disease stages, and other strawberry diseases; • compare U‑Net with newer segmentation models (Swin‑ UNet, TransUNet, Mask2Former) for gauge accuracy gains; • explore transfer learning using pretrained models (Plant- CLEF, Agrivision) to reduce labeled data requirements; • use multispectral time series (every 3–5 days) for early detection before visible symptoms appear.

## Conclusion

The study confirmed the feasibility of automated detection of leaf spot in garden strawberries using multispectral UAV imagery. The deep learning U‑Net model achieved an overall classification accuracy of 96.04 %, significantly surpassing the performance of the Random Forest model (76.64 %) and the U‑Net model trained solely on RGB images (94.14 %). This demonstrates that the U‑Net architecture equipped with attention mechanisms and residual connections, together with the use of the near‑infrared (840 nm) and red‑edge (730 nm) channels, is essential for diagnosing diseases with scattered small lesions, where traditional texture features fail.

The practical significance of this work lies in the ability to generate detailed infection distribution maps for targeted fungicide application.

## Conflict of interest

The authors declare no conflict of interest.
